# Comparison of immune responses to respiratory syncytial virus in infancy, childhood, and adulthood using an in vitro model of human respiratory infection

**DOI:** 10.1093/immhor/vlae010

**Published:** 2025-01-24

**Authors:** Christiana Smith, Kaili Curtis, Adrianne Bonham, Shea Boyer, Laurel Lenz, Adriana Weinberg

**Affiliations:** Section of Infectious Diseases and Epidemiology, Department of Pediatrics, University of Colorado, Aurora, CO, United States; Section of Infectious Diseases and Epidemiology, Department of Pediatrics, University of Colorado, Aurora, CO, United States; Section of Infectious Diseases and Epidemiology, Department of Pediatrics, University of Colorado, Aurora, CO, United States; Section of Infectious Diseases and Epidemiology, Department of Pediatrics, University of Colorado, Aurora, CO, United States; Departments of Immunology and Microbiology, University of Colorado, Aurora, CO, United States; Section of Infectious Diseases and Epidemiology, Department of Pediatrics, University of Colorado, Aurora, CO, United States; Departments of Medicine and Pathology, University of Colorado, Aurora, CO, United States

**Keywords:** cord blood, infant, innate immune response, respiratory cell model, respiratory syncytial virus

## Abstract

Respiratory syncytial virus (RSV) is a major contributor to morbidity and mortality in infants. We developed an in vitro model of human respiratory infection to study cellular immune responses to RSV in infants, children, and adults. The model includes human lung epithelial A549 cells or human fetal lung fibroblasts infected with a clinical strain of RSV at a multiplicity of infection of 0.3, cocultured with human cord blood mononuclear cells (CBMCs) or peripheral blood mononuclear cells (PBMCs). Mononuclear cells were collected at multiple ages ranging from birth to adulthood. After 20 h of incubation, flow cytometry was used to measure CBMC/PBMC responses to RSV. A549s were more permissive to RSV and when infected produced more CCL5, CCL11, and CXCL9; less CSF-3, CXCL10, interleukin (IL)-1α, IL-1RA, and IL-6; and similar CCL2, CCL3, CCL4, CCL7, CXCL1, CXCL11, IL-1β, IL-7, IL-8, and tumor necrosis factor α compared with fibroblasts; A594s were used for subsequent experiments. CBMCs/PBMCs upregulated multiple markers of activation, maturation, and degranulation upon exposure to RSV-infected A549s. Interferon γ expression in natural killer, CD4, and CD8 cells and CD107a expression in natural killer cells showed a gradual increase from infancy to adulthood. IL-12 expression in dendritic cells and monocytes was highest in adult PBMCs. Our in vitro model of human RSV infection recapitulated the expected bias away from T helper 1 and effector responses to RSV infection in infancy and revealed changes in innate and adaptive RSV-specific cellular immune responses over time.

## Introduction

Respiratory syncytial virus (RSV) causes substantial global morbidity and mortality among children under age 5 yr.^1^ The majority of children who are hospitalized with RSV are previously healthy term infants younger than 3 mo of age, whereas older children and adults who acquire RSV typically experience less severe disease.^2–5^ The decreasing morbidity observed with increasing age at RSV infection can be attributed both to gradual maturation of the immune system and cumulative RSV exposure resulting in immune memory. However, immune memory only provides partial protection; humans remain susceptible to reinfection with antigenically similar strains of RSV throughout life.

Neonates rely heavily on maternal antibodies and secondarily on innate immune responses for protection against RSV, given their developing adaptive immune system. However, even innate immune defenses in neonates are limited in their ability to control RSV infection.^6–8^ RSV primarily infects the superficial epithelial cells of the upper and lower respiratory tract, as well as type I alveolar cells. Toll-like receptor stimulation in respiratory epithelial cells leads to a cascade of proinflammatory cytokines and chemokines that recruits innate immune cells including natural killer (NK) cells, dendritic cells (DCs), monocytes, and neutrophils.^8–10^ These innate cells play several crucial roles in the control of RSV infection, including clearance of infected cells, production of cytokines and other substances with antiviral activity, and induction of adaptive immune responses.^11^ While numerous platforms are available to measure humoral and cellular adaptive immune responses to RSV infection (eg, enzyme-linked immunosorbent assay [ELISA], enzyme-linked immunosorbent spot, neutralizing antibody assays), assays to measure cellular innate immune responses are less available due to technical challenges.

We developed an in vitro model involving a coculture of human cord or peripheral blood mononuclear cells with respiratory epithelial cells. This coculture design allows the RSV to replicate in the respiratory epithelial cells, mimicking infection in vivo, and allows for cell-to-cell crosstalk between epithelial and immune cells. Using this model, we reveal differences in RSV-specific innate and adaptive cellular immune responses among neonates, toddlers, and adults.

## Materials and methods

### A549 and fibroblast cultures

We obtained the A549 human alveolar epithelial cell line from American Type Culture Collection. Fetal lung fibroblasts were obtained from the Diagnostic Virology laboratory of the Department of Pediatrics at the University of Colorado School of Medicine. Both cell types were maintained in minimum essential medium (MEM) containing GlutaMAX (Gibco; ref. 41090-036) supplemented with 10% fetal bovine serum (FBS) (GemCell; cat.100–500). Cells were cultured under standard conditions (37 °C with 5% CO_2_) and kept in monolayers up to 90% confluence. A549s were used up to passage 120 and fibroblasts up to passage 14.

### RSV quantification, amplification, and isolation

RSV was originally isolated from a clinical sample at the University of Colorado Hospital. The virus was expanded in human lung fibroblast tissue culture and infectivity was assessed by titration. We performed five 10-fold dilutions (10^−1^ to 10^−5^) of the stock virus into MEM with 10% FBS. We inoculated R-Mix Too Shell Vials (Quidel; cat. 97-0102) with each dilution in duplicate and centrifuged the shell vials at 700 × *g* at 25 °C for 60 min, then incubated at 37 °C with 5% CO_2_ for 48 h. After incubation, we fixed, stained, and mounted the coverslips according to the Quidel D^3^ Ultra DFA RSV Reagent Set (cat. 01-010003.v2) protocol. We counted the fluorescent foci positive for RSV using a fluorescent microscope. Our final titer of RSV was calculated to be between 4 and 5 × 10^5^ fluorescent focus units/mL.

Seed virus stocks were prepared by passaging the virus in either fetal lung fibroblasts or A549 cells. Cells were seeded into 75 cm^2^ tissue culture flasks and cultured as described previously until they reached 70% to 80% confluence. Media was removed from the flasks and 3 mL stock virus plus 10 mL virus growth media (MEM with 2% FBS) was added to the flasks (for a target multiplicity of infection [MOI] of ∼0.3 fluorescent focus units/cell). The flasks were placed on a plate shaker at 50 rpm at 37 °C for 1 hour, then 25 mL of virus growth media was added and the flasks were incubated as described previously for 3 to 5 d until a cytopathic effect (CPE) was observed. The culture supernatant containing RSV was collected from each flask and pooled. Adherent cells were rinsed twice with phosphate-buffered saline (PBS), then removed from the flasks using 2 mL of 0.25% trypsin (Gibco; cat. 25200-056) and added to the pooled supernatant. The supernatant was clarified by centrifugation at 500 × *g* at 25 °C for 10 min. Approximately 5 mL of supernatant was used to resuspend the cell pellet. We sonicated the resuspended cells for 30 s at 25 °C to release any membrane-bound virus, then centrifuged again at 500 × *g* for 10 min and added the supernatant to the pooled viral stock. RSV stock was aliquoted and stored at −80°C.

### A549 and Fibroblast infection with RSV

A549 epithelial cells or fibroblasts were seeded into sterile flat-bottomed, polystyrene 24-well tissue culture plates (Corning; ref. 3524) at 100,000 cells/well. RSV was immediately added at an MOI of ∼0.3 in a total volume of 500 µL MEM with 10% FBS per well. After incubation for 24 h at 37 °C, 5% CO_2_, media was changed to fresh MEM with 10% FBS and cells were incubated for a further 48 to 72 h to establish productive infection.

### Cytokine/chemokine measurement in cell cultures

A549s or fibroblasts were grown in 24-well plates as described previously. Duplicate wells of each cell type were infected with RSV or mock infected. Media was replaced every 24 h for 5 d and the supernatant removed, snap-frozen, and stored at −80°C. Cytokine and chemokine concentrations in supernatant were measured using custom multiplex electrochemiluminesence microarrays (Meso Scale Diagnostics). Measurements represent production of each analyte in the previous 24 h.

### Participant information

Adult PBMCs were obtained from healthy anonymous adult volunteers who donated blood at Children’s Hospital Colorado. Cord blood mononuclear cells (CBMCs) and infant/child PBMCs were obtained from healthy pregnant women, infants, and children enrolled in research protocols at the University of Colorado and/or Children’s Hospital Colorado. Newborn infants (who donated either cord blood or peripheral blood in the first 48 h of life) were excluded if they were born at <37 wk gestational age; were a product of a multiple gestation; had any congenital anomaly; were born to a mother with active malignancy, autoimmune condition, or acute or chronic active infection during pregnancy (eg, HIV, hepatitis B or C, SARS-CoV-2, syphilis, tuberculosis); or were exposed to a delivery complication such as chorioamnionitis or severe fetal distress requiring operative delivery. Infants 12 to 18 mo of age at enrollment were excluded if they had any congenital or acquired immunodeficiency, a known genetic disorder, a chronic infection (eg, HIV, hepatitis B or C, syphilis, tuberculosis), or malignancy, or had received any immune-modulating medications (eg, corticosteroids, immune globulin). Cord blood was collected by needle aspiration from placental vessels after the cord was clamped. Peripheral blood was collected via venipuncture. Enrollment in research and the use of samples for this study was approved by the Colorado Multiple Institutional Review Board (protocols #19-1911, 23-0175), and all participants or caregivers provided written informed consent.

### Anti-RSV IgG ELISA

Whole blood was processed within 8 h of collection and plasma was aliquoted and cryopreserved at −80°C until use. Qualitative anti-RSV IgG ELISA (IBL America; IB79887) was used to determine seropositivity to RSV (cutoff of 10 ELISA units) among infants who were 12 to 18 mo of age at the time of the blood draw.

### PBMC/CBMC isolation

We isolated PBMCs/CBMCs from the buffy coat of whole blood using Ficoll-Histopaque (Sigma-Aldrich; cat. H8889) gradient centrifugation and cryopreserved the cells in FBS containing 10% dimethyl sulfoxide (Sigma-Aldrich; D2660). Cells were stored at −150°C until use.

### Coculture of PBMCs/CBMCs with respiratory cells

After thawing, PBMCs/CBMCs were washed twice with MEM containing GlutaMAX supplemented with 10% FBS and 2 µL/mL Benzonase (Sigma-Aldrich; 70746-3) and then resuspended in Xvivo media containing gentamicin (Lonza; cat. 04-418Q). Cell counts and viability were obtained using a Guava easyCyte 5HT System (Cytek Biosciences) instrument and samples with a minimum of 80% viability and 3,000,000 viable cells were included. After removal of the media from each well containing A549 or fibroblast cultures, PBMCs/CBMCs were added at a density of 1.5 × 10^6^ cells in 500 µL per well. Cells from each donor were divided between one RSV-infected and one mock-infected well. One well of A549s or fibroblasts in both the RSV-infected and mock-infected conditions received no PBMCs/CBMCs as a control; media was replaced in those wells with Xvivo. Cocultures were incubated at 37 °C, 5% CO_2_ for 20 h.

### Flow cytometry

For cell cultures intended for flow cytometry, A549s or fibroblasts were stained prior to seeding. Cells were resuspended in PBS at 10^6^ cells/mL and stained using a 1:10 dilution of Cell Trace Violet (Invitrogen; cat. C34557) for 20 min at room temperature. After incubation, cells were centrifuged at 500 × *g* at 25 °C for 10 min and the pellet was resuspended in MEM with 10% FBS. Stained A549s or fibroblasts were then seeded into 24-well plates, RSV infected or mock infected, and cocultured with PBMCs/CBMCs as described previously.

For the final 4 h of incubation, the secretion inhibitors brefeldin A (Sigma-Aldrich; cat. B7651) and monensin (BD Biosciences; cat. 554724) were added at a final concentration of 5 µg/mL each. Additionally, 5 µL of CD107a conjugated to BV711 (BioLegend; cat. 328640) was added to all wells containing PBMCs/CBMCs.

At the end of incubation, PBMCs/CBMCs and supernatants were harvested via pipette aspiration. Accutase (Millipore; cat. SCR005) was used per the manufacturer’s instructions to remove adherent cells, which were combined with PBMCs/CBMCs and supernatant in 15 mL conical tubes. Wells were scraped and washed twice with PBS and each wash added to the 15 mL conical. Samples were centrifuged at 500 × *g* for 5 min, media was removed, and cells were resuspended in PBS. Cells were transferred to a 96-well round-bottom polypropylene plate for staining.

**Figure 1.**
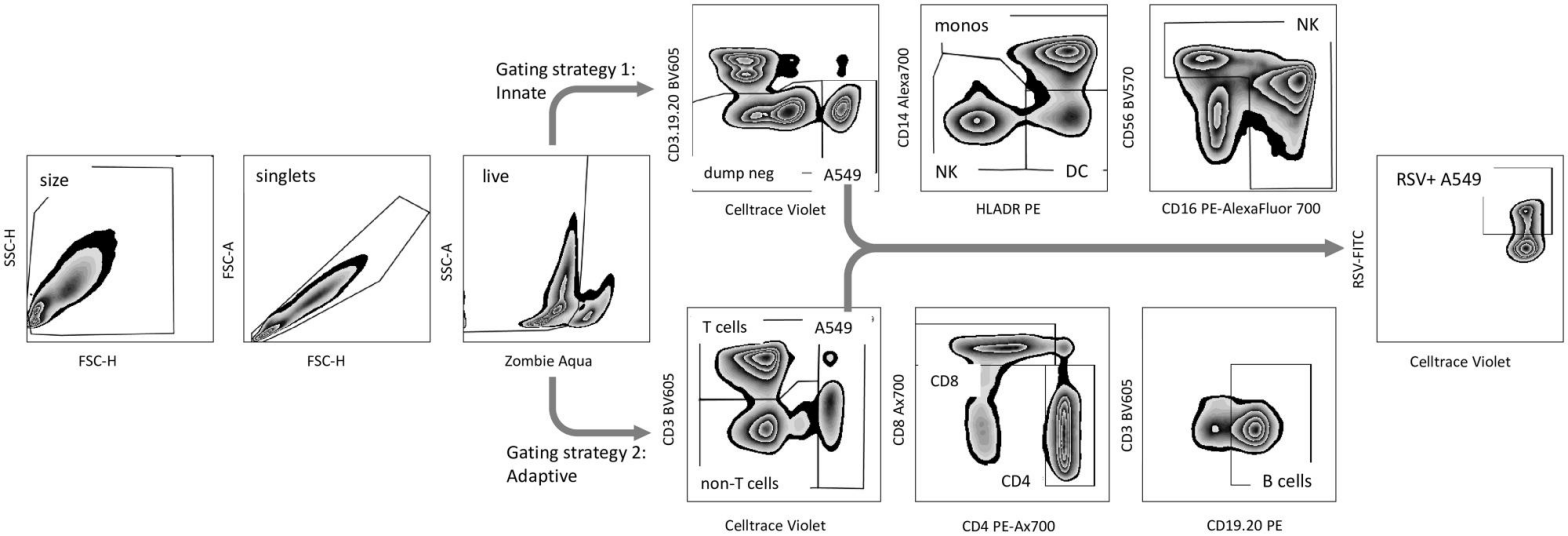
Representative gating strategy. CBMCs or PBMCs were incubated for 20 h with RSV-infected or mock-infected A549 respiratory epithelial cells. All cells were subsequently removed from the coculture model and stained with the monoclonal antibodies shown. Flow minus one was used to determine gate placement. FSC-A = forward scatter area; FSC-H = forward scatter height; SSC-A = side scatter area; SSC-H = side scatter height.

Cells were stained with the live/dead cell discriminator Zombie Aqua (BioLegend; cat. 423101) prior to being incubated with an Fc receptor block (BioLegend; cat. 422302) according to the manufacturer’s instructions. Cells were stained extracellularly with monoclonal antibodies against the following: CD3, CD19, and CD20 BV605; CD56 BV570; CD14 Alexa 700; CD16 PE-Cy5; HLADR PE; CD83 PE-Cy7; CD8 Alexa 700 (BioLegend); CD19 and CD20 PE (BD Pharmingen); CD4 PE-Alexa700 (Invitrogen); and PD-L1 BV650 (BD Horizon). Cells were fixed with FACS lysing solution (BD; cat. 349202) and permeabilized with FACS permeabilizing solution (BD; cat. 347903) and stained for intracellular markers with monoclonal antibodies against the following: RSV FITC (Invitrogen); interleukin (IL)-12 APC; IL-13 APC (BD Pharmingen); Perforin APC-Cy7; interferon γ (IFNγ) BV786; and granzyme B PE-CF594. Sample acquisition was performed using a NovoCyte Quanteon 4025 flow cytometer (Agilent) and analyzed using FlowJo version 10.8.1 software (BD). For the gating strategy, see Fig. 1. Markers of activation in PBMCs are reported as fold change in the proportion of parent population expressing the marker or in median fluorescence intensity between RSV-infected and mock-infected cultures.

### Cell enrichment experiments

We isolated NK cells, DCs, and monocytes from healthy adult PBMCs using STEMCELL Technologies kits: EasySep Human NK Cell Isolation Kit (cat. 17195), EasySep Human Monocyte Isolation Kit (cat. 19319), and EasySep Human Pan-DC Pre-Enrichment Kit (cat. 19211). The purity of the isolated NK cells was >90%, DCs was >80%, and monocytes was >95%. Enriched NK cells were then added to the model at a density of 100,000 cells in 500 µL per well and incubated as previous; responses were compared with nonenriched NK cells added to the model along with other PBMCs from the same donor at a density of 1.5 × 10^6^ cells in 500 µL per well. Enriched DCs and monocytes were combined and then added to the model at a density of 150,000 cells in 500 µL per well and incubated as previous; responses were compared with nonenriched antigen-presenting cells added to the model along with other PBMCs from the same donor at a density of 1.5 × 10^6^ cells in 500 µL per well.

### Statistical analysis

All statistical analyses were performed in GraphPad Prism, version 10.2.1 (GraphPad Software). *P *< 0.05 was considered significant. In experiments that measured a panel of cytokines and chemokines in the supernatant of A549 and fibroblast cultures, we used 2-way repeated-measures analysis of variance to determine whether the change in cytokine/chemokine concentrations over time differed between respiratory cell types. In experiments that measured the expression of markers of activation in CBMCs/PBMCs after exposure to RSV-infected versus mock-infected respiratory epithelial cells, we used Mann-Whitney, Kruskal-Wallis, or Friedman tests to compare expression between different age groups or conditions.

## Results

### A549 epithelial cell and fetal lung fibroblast cultures respond differently to RSV infection

A549s or fibroblasts were grown in 24-well plates and infected with RSV at an MOI of ∼0.3. After 48 to 72 h, both cell types demonstrated a CPE, which increased over time, but the CPE had a different appearance in each cell type (Fig. S1).

After harvesting the A549s or fibroblasts, RSV-infected cells were identified by staining intracellularly with anti-RSV nucleocapsid antibody conjugated to FITC. The proportion of cells that stained RSV+ increased as the cells were infected at a higher MOI (Fig. S1). Fibroblasts were noted to be less permissive to RSV infection (∼10%–25% RSV+ cells) than A549s (∼30%–60% RSV+ cells) at the same MOI of 0.3.

**Figure 2.**
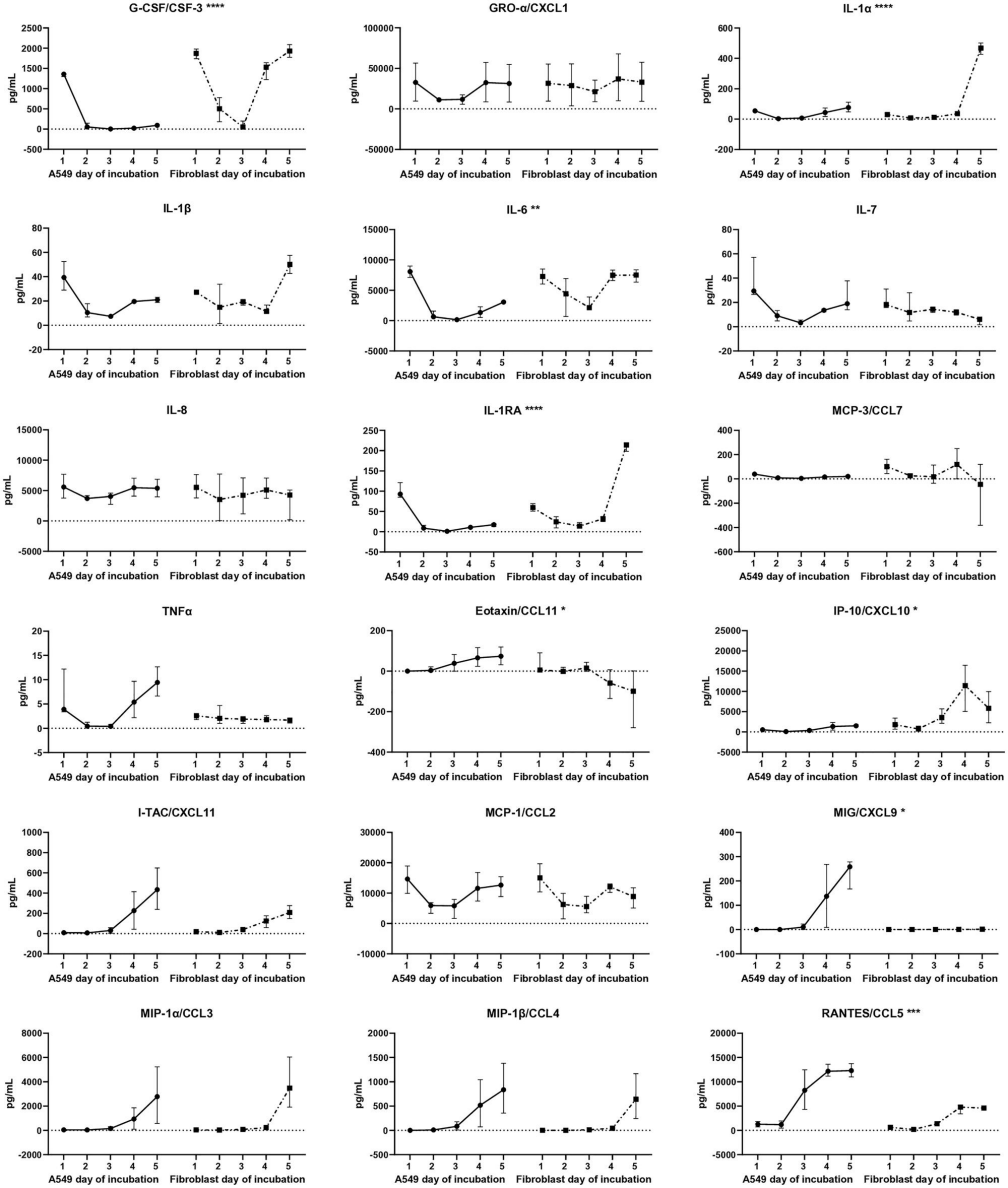
Cytokine and chemokine concentrations measured in cell culture supernatants from A549s or fibroblasts every 24 h after RSV infection. Values are represented as median concentration (pg/mL) and interquartile range with concentrations in mock-infected cultures subtracted at each time point. The culture medium was almost completely replaced on each day of the assay, such that data represent cytokine secretion per day as opposed to cumulative secretion. Assays were performed in duplicate in 2 independent experiments. Differences between cell types in the median concentration change over time are indicated by asterisks: *P = 0.01 to 0.05, **P = 0.001 to 0.01, ***P = 0.0001 to 0.001, and ****P < 0.0001. G-CSF = granulocyte colony-stimulating factor; TNFα = tumor necrosis factor α.

Human respiratory cells are known to produce many cytokines and chemokines that play an important role in attracting and maintaining immune cells at the site of infection.^12–18^ We measured a panel of cytokines and chemokines in the supernatant of RSV-infected and mock-infected A549 and fibroblast cultures to compare the patterns of expression between these cell types each day over the 5 d after infection by removing and replacing the culture medium while verifying that cells were still present in each well with an inverted microscope. While low concentrations of cytokines and chemokines were measured in the mock-infected cultures (Fig. S2), concentrations were higher in RSV-infected cultures (Fig. 2) and several cytokines and chemokines, including CSF-3, IL-1α, IL-6, IL-1RA, CCL11, CXCL10, CXCL9, and CCL5, showed differences in concentration over time between A549 and fibroblast cultures. Most differences in concentrations between the A549 and fibroblast cultures were observed on days 4 or 5 post–RSV infection.

### Coculture of PBMCs with RSV-infected respiratory cells attenuates the RSV infection and activates the PBMCs

We investigated the effect of coculture of RSV-infected A549 cells with PBMCs from healthy adult donors. The addition of PBMC to the A549 cultures reduced the proportion of RSV-infected A549 cells. As the number of PBMCs or the duration of coculture increased, the proportion of RSV-infected A549 cells decreased in a dose-dependent fashion (Fig. S3).

**Figure 3.**
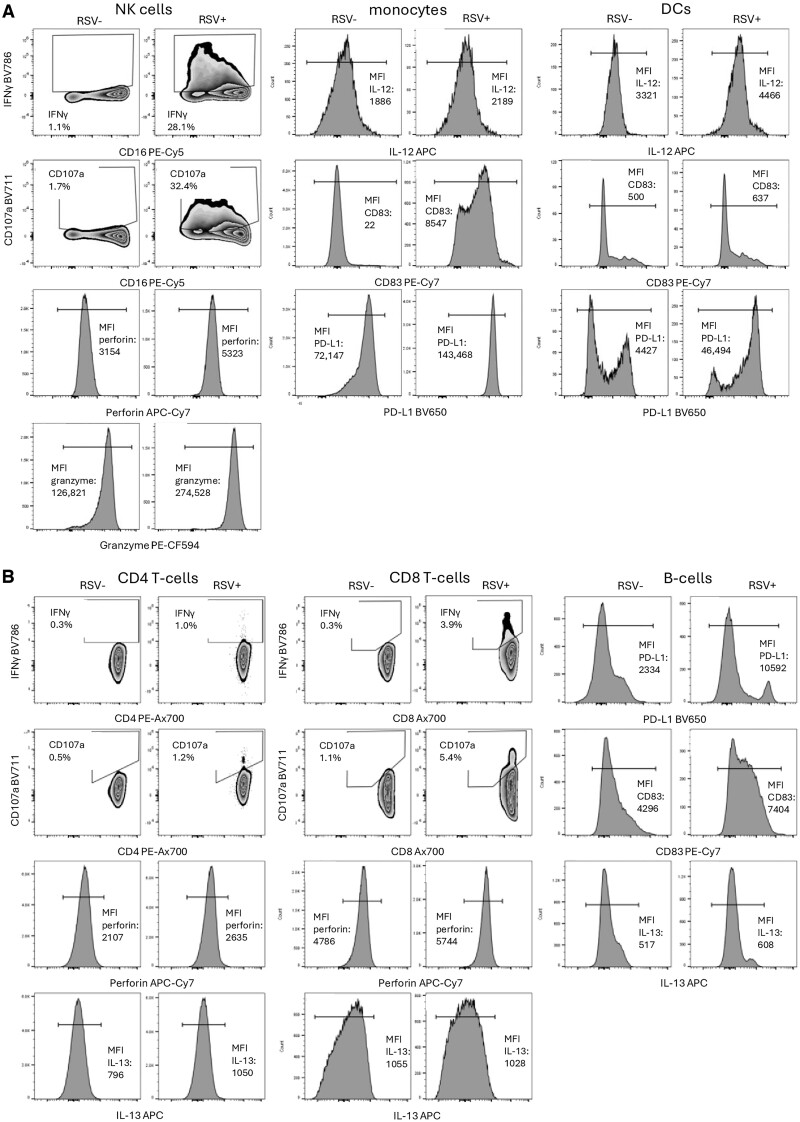
Markers of activation, maturation, and degranulation in PBMCs upon exposure to RSV-infected respiratory cells. CBMCs or PBMCs were incubated for 20 h with RSV-infected or mock-infected A549 respiratory epithelial cells. All cells were subsequently removed from the coculture model and stained with the monoclonal antibodies shown. Flow minus one was used to determine gate placement. In plots depicting median fluorescence intensity (MFI), the MFI calculation was performed on the gated population. (A) Markers in innate immune cells. (B) Markers in adaptive immune cells.

PBMCs upregulated multiple markers of activation, maturation, and degranulation upon exposure to RSV-infected respiratory cells in the coculture model (Fig. 3). In this model, T cells expressed IFNγ, CD107a, and perforin but did not express significant quantities of IL-13; B cells expressed IL-13, PD-L1, and CD83; and NK cells expressed IFNγ, CD107a, perforin, and granzyme B. Antigen-presenting cells, including DCs and monocytes, expressed PD-L1, IL-12, and CD83. All populations of PBMCs appeared to take up RSV nucleocapsid to some degree, with the highest proportion of monocytes staining positive for RSV nucleocapsid (up to 50%) and the lowest proportion of T cells staining positive (<2%). Importantly, there was little to no evidence of PBMC activation, or RSV staining, upon exposure to mock-infected respiratory cultures.

We evaluated whether PBMC responses differed when exposed to RSV-infected A549 cells versus fibroblasts and when exposed on day 3 versus day 4 after RSV infection, as the varying concentrations of cytokines and chemokines produced by the RSV-infected respiratory cells on different days of infection (as shown in Fig. 2) may potentially impact PBMC activation. We identified very few differences between PBMC responses to RSV in these 4 coculture conditions, suggesting that PBMC activation was primarily a response to the viral infection itself, rather than a response to the cytokines produced by the infected cells (data not shown). For all subsequent experiments, we used A549 cells and added PBMCs to the coculture on day 3 after RSV infection.

We used magnetic bead–based cell enrichment kits to isolate NK cells, DCs, and monocytes from healthy adult PBMCs. We compared the responses of NK cells to RSV-infected A549s when added to the model in isolation versus together with all PBMCs from the same participant. Separately, we compared the responses of antigen-presenting cells (DCs and monocytes) to RSV-infected A549s when added to the model in isolation versus together with all PBMCs from the same participant. As shown in Fig. S4, NK cell responses (expression of IFNγ, CD107a, and granzyme B, with a trend for expression of perforin) were significantly diminished when added to the model without other PBMC types. In contrast, antigen-presenting cell responses demonstrated no differences when added to the model alone versus with other PBMC types.

### The responses to RSV-infected epithelial cells change from birth to adulthood in multiple immune cell types

We used CBMCs from healthy mother-infant dyads and PBMCs obtained from healthy newborns in the first 48 h of life, healthy children at 12 to 18 mo of age, and healthy adults to demonstrate the natural progression of innate and adaptive immune responses to RSV across the lifespan. The demographics of the infants and children are described in Table S1. Demographic information was not available for the anonymous adult blood donors. We considered all newborn infants to be unexposed to RSV infection and all adult donors to have been exposed to RSV. We used ELISA to determine whether children at 12 to 18 mo of age were RSV seronegative or RSV seropositive.

**Figure 4.**
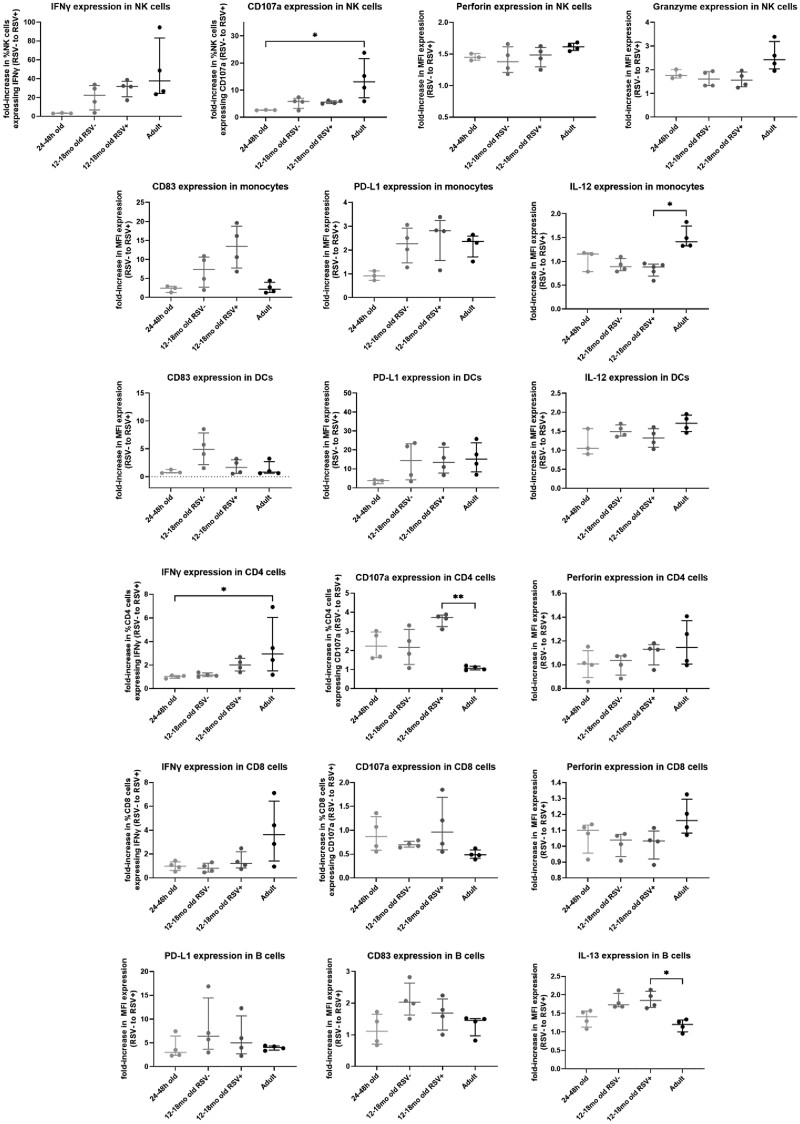
Fold change in expression of markers of activation, maturation, and degranulation among PBMCs from participants of different ages after exposure to RSV-infected versus mock-infected respiratory epithelial cells. n = 4 participants were included at each age; all samples were run in the same experiment. Significant differences are indicated by asterisks: *P = 0.01 to 0.05 and **P = 0.001 to 0.01.

Figure 4 shows the expression of markers of activation, maturation, and degranulation among several types of innate and adaptive immune cells after exposure to RSV-infected respiratory epithelial cells at multiple ages from birth to adulthood. Given the small numbers of samples tested, few differences met statistical significance; however, there were several important trends. Several markers (eg, IFNγ in NK, CD4, and CD8 cells; CD107a in NK cells) showed an expected gradual increase from 24 to 48 h of life to adulthood. For several markers, the highest expression was observed in adults (IFNγ in NK cells, CD4, and CD8 cells; perforin in CD4 and CD8 cells; granzyme in NK cells; IL-12 in DCs and monocytes); for other markers, adults had lower expression than the younger-aged participants (CD107a in CD4 and CD8 cells, IL-13 in B cells, CD83 in monocytes). Some markers appeared to reach their peak expression by 12 to 18 mo of age (CD83 in B cells, monocytes, and DCs; PD-L1 in B cells, monocytes, and DCs; IL-13 in B cells). There were no significant differences in responses between 12- to 18-mo-old infants who were RSV seronegative versus RSV seropositive.

In several cell types, we identified high expression of activation markers in CBMCs compared with that seen in PBMCs collected from infants at 24 to 48 h of life (eg, IFNγ and granzyme in NK cells, IL-12 in DCs, perforin in CD8 cells, IL-13 in B cells). This finding indicates that there are unique features of the responses from CBMCs versus those from infant PBMCs collected at 24 to 48 h of life. While the mechanisms contributing to these differences are not known, their existence argues that the common practice of using CBMCs as a proxy for newborn PBMCs is flawed and suggests that infant PBMCs are a more appropriate set point against which to compare the responses in older participants (Fig. S5).

## Discussion

Our in vitro model of RSV infection demonstrated differences in innate and adaptive RSV-specific cellular immune responses between individuals of different ages that represent development of the immune system and increasing exposure to RSV over time. Whereas several studies have compared immune responses with RSV between CBMCs and adult PBMCs,^19–23^ this is the first study, to our knowledge, to compare immune responses with RSV at 3 time points between 24 and 48 h of life and adulthood using an in vitro model of RSV infection. Our model recapitulated the expected bias away from T helper 1 and effector responses to RSV infection in infancy, with low expression of IL-12, IFNγ, perforin, and granzyme in early life that increased in adulthood.^7^ An additional expected finding was the predisposition toward T helper 2 responses to RSV infection in infancy/early childhood, with higher expression of IL-13 in early life that declined in adulthood.^24^ Importantly, innate immune responses to RSV (eg, IL-12 expression in DCs and monocytes, IFNγ expression in NK cells) were impaired to a similar degree as adaptive immune responses among PBMCs from infants and young children in this model. IFNγ and IL-12 are heavily epigenetically regulated in infancy, so this pattern is expected.^6^ The early immune response to RSV infection is highly dependent on a pathway that includes production of IL-12 by antigen-presenting cells, which triggers the release of IFNγ by NK cells.^6^^,^^7^ The importance of the crosstalk between antigen-presenting cells and NK cells on NK cell effector function was demonstrated by the experiments in we added isolated NK cells to the model in the absence of other cell types. In summary, this model recapitulated the biased innate immune responses that likely contribute to more severe RSV-associated morbidity in infants as compared with older children and adults.

We identified that several markers of activation and degranulation were higher in cord blood than at 24 to 48 h of life, which we hypothesize is a result of the inflammatory response associated with the stress of birth. In agreement with previous reports, our data suggest that CBMC responses ex vivo are not representative of actual neonatal immune responses in vivo, and that CBMC responses should not be used as a proxy for neonatal immune responses.^25^

The greatest burden of RSV morbidity is in infants under 3 mo of age, suggesting a period of immunological vulnerability.^2–5^ However, our data demonstrate that dysfunctional immune responses to RSV persist beyond the neonatal period. The average age at RSV-associated hospitalization has increased since the COVID-19 pandemic, likely as a result of decreased early exposure to RSV during periods of nonpharmaceutical prevention measures (eg, masking, social distancing).^26^^,^^27^ This suggests that not only immune development, but also cumulative RSV exposure are needed to decrease the morbidity associated with RSV infection. In 2023, 2 new RSV prevention tools became available to prevent RSV-associated morbidity in infants: a maternal RSV vaccine (Pfizer Abrysvo) and a monoclonal antibody administered to infants (nirsevimab).^28–30^ Importantly, neither of these prophylactic agents depends on the neonate to generate an immune response to RSV; rather, they protect infants by providing them with passive anti-RSV antibodies. Infant candidate RSV vaccines have not reached phase 3 clinical trials due to unsatisfactory phase 1 and 2 results.^31–35^ The numerous limitations in the neonatal immune response to RSV, as illustrated by our model, shed light on the reasons for the poor success of RSV vaccines to date in this population.^36^^,^^37^

Most prior in vitro models designed to study human RSV infection have used well-differentiated respiratory epithelial cells, but did not include human immune cells, or have exposed human immune cells to inactivated or live RSV in the absence of respiratory epithelial cells.^16^^,^^20^^,^^38^^,^^39^ Our model is one of few in the literature that included a coculture of RSV-infected respiratory epithelial cells with PBMCs.^23^^,^^40–42^ This design is important because it allows the RSV to replicate in the respiratory epithelial cells, which mimics infection in vivo. In our model, the proportion of epithelial cells that stain positive for RSV can be used as a proxy for the PBMCs’ ability to suppress viral replication. However, it is unclear whether the PBMCs are killing or resolving the infection in the RSV-infected respiratory epithelial cells. The inclusion of the respiratory epithelial cells in the model is also important to simulate the cell-to-cell crosstalk that occurs between epithelial and immune cells during RSV infection. A limitation of our model is that the ideal ratio of PBMCs to respiratory epithelial cells that will accurately replicate the in vivo environment is unknown, as this ratio shows significant variability over the course of an infection as peripheral immune cells are recruited into the lungs.^7^^,^^8^^,^^18^

We found that either the A549 cell line or fetal lung fibroblasts worked well in this model, as they were both permissive to RSV infection and tolerated media that was optimal for culturing PBMCs. In our experience, primary bronchial epithelial cells and commercially available small airway epithelial cells require special media that contains steroids, insulin, growth factors, and other additives that may artificially stimulate PBMCs in a similar model.^16^^,^^42^ The A549 and fetal lung fibroblast cultures responded differently to RSV infection in our model, with different CPE visible on gross inspection and some differences in expression of cytokines and chemokines. However, the overall pattern of cytokines and chemokines expressed by both A549s and fetal lung fibroblasts in this model were similar to those identified in the respiratory tract of human infants with severe RSV infection, and to those measured in other in vitro models of RSV infection.^16^^,^^18^^,^^43–45^ An outstanding question that deserves further exploration is whether pediatric respiratory epithelial cells behave differently in in vitro models than adult respiratory epithelial cells in terms of permissiveness to viral replication and cytokine/chemokine production.^46^

Limitations of our model include that it is a simplified version of a human lung, in that it only contains 2 cell types (respiratory epithelial cells and PBMCs), whereas in vivo, other cell types such as stromal cells and endothelial cells likely contribute to the immune response to infection. In addition, the respiratory epithelial cells in our model were not differentiated with an air-liquid interface to induce polarized, ciliated cells. While a few investigators have been successful in coculturing PBMCs with well-differentiated, ciliated respiratory epithelial cells in an air-liquid interface, we have found that such a model limits cell-to-cell contact between PBMCs and epithelial cells, or requires resubmerging the apical surface of the respiratory cells in media to encourage PBMC migration, which is not physiologic.^15^^,^^23^^,^^40–42^ On the other hand, the ample cell-to-cell contact in our model does not allow for dissection of whether the PBMC activation that we observed was a result of direct contact or soluble factors released by infected epithelial cells. We only tested our model with a single clinical isolate of RSV, and different viral strains are known to result in different cytopathogenesis.^47^ However, we believe that a clinical isolate of RSV is more appropriate to use in this context than a laboratory strain that has been adapted to immortal cell lines. We included small numbers of individuals at each age, resulting in low power to identify differences between immune responses in each age group, although trends were apparent. We were not able to include PBMCs from infants between 24 and 48 h of life and between 12 and 18 mo of life, nor between 12 and 18 mo and adulthood, but future studies could further explore the nuanced timing of changes in the immune response to RSV over the lifespan. Finally, the use of cryopreserved PBMCs limited our ability to evaluate granulocytes in this model, which is unfortunate because neutrophils play a significant role in the response to RSV infection.^23^^,^^48^ Future studies could use this model to evaluate the role of granulocytes and other immune cells such as γδ T cells or mucosal-associated invariant T cells in RSV responses. Future studies could also explore the mechanism of immune responses in this model (eg, using inhibitory agents to block the activity of immune mediators, receptors, and/or viral antigens). Despite these limitations, here we present a simple, in vitro model of human RSV infection that can be used to compare RSV-specific innate and adaptive cellular immune responses to RSV between individuals of different ages.

## Supplementary Material

vlae010_Supplementary_Data

## Data Availability

The data underlying this article will be shared on reasonable request to the corresponding author.
