# Supplementary material for: Comparison of immune responses to respiratory syncytial virus in infancy, childhood, and adulthood using an in vitro model of human respiratory infection
Source: Immunohorizons. 2025 Jan 24;9(2):vlae010. doi: 10.1093/immhor/vlae010 (PMC11841974; doi:10.1093/immhor/vlae010)
Supplement: vlae010_Supplementary_Data [file vlae010_supplementary_data.pdf]

Supplementary Figure 1.

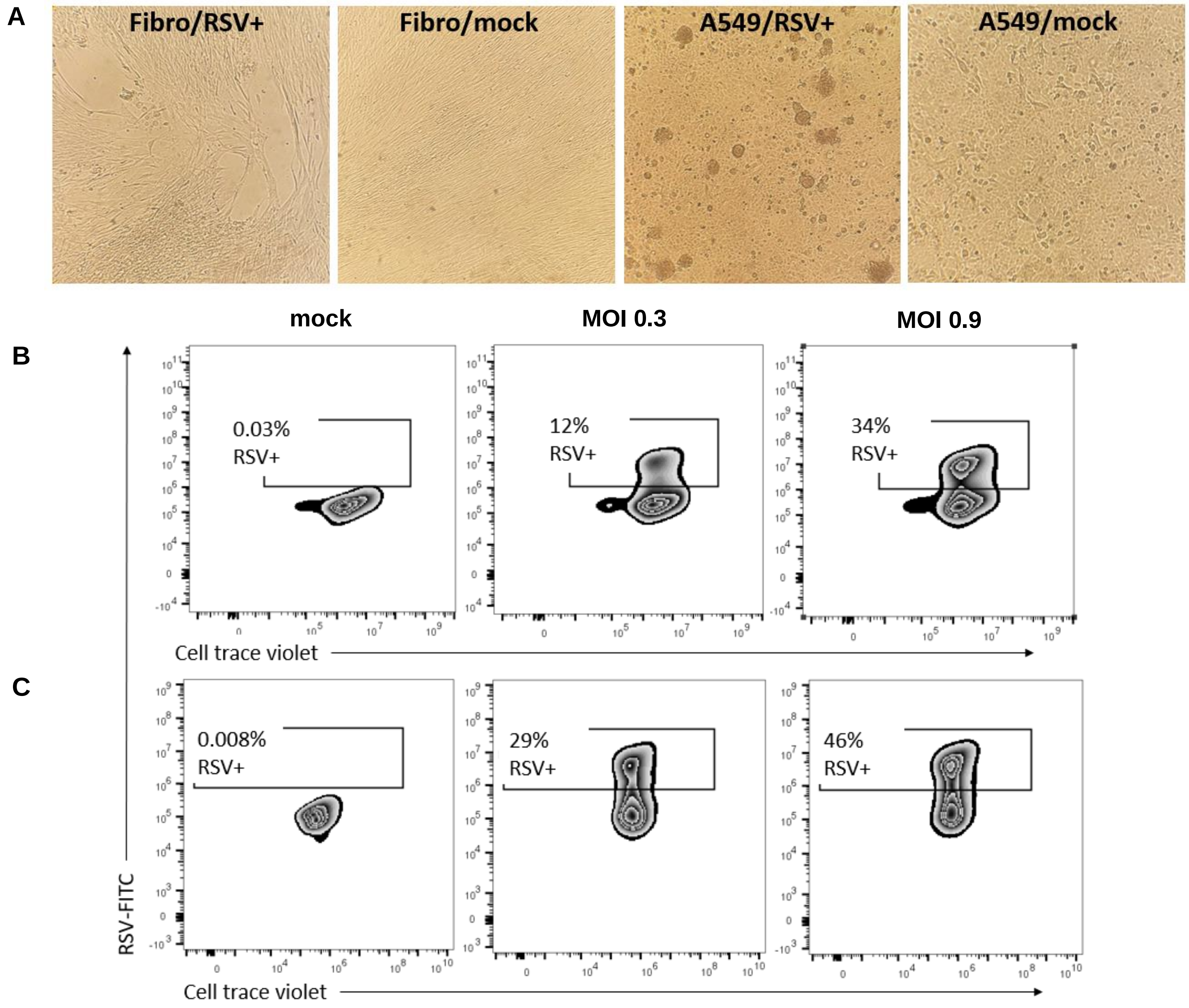

Supplementary Figure 1. RSV establishes productive infection in respiratory epithelial cells. A) Cytopathic effect of RSV in fetal lung fibroblasts and A549s. Fibroblasts or A549s were grown in 24 well plates and infected with a clinical strain of RSV at MOI 0.3 or mock infected. Micrographs of a representative field for each cell type are shown at 72 hours post infection. Original magnification, x10. B) The proportion of fibroblasts or C) A549 epithelial cells that stain positive for RSV increases with a higher MOI of RSV infection. Fibroblasts or A549 epithelial cells were infected with RSV at varying MOI, then harvested at 72 hours post infection and stained intracellularly with anti-RSV nucleocapsid antibody conjugated to FITC to identify RSV-infected cells.

Supplementary Figure 2

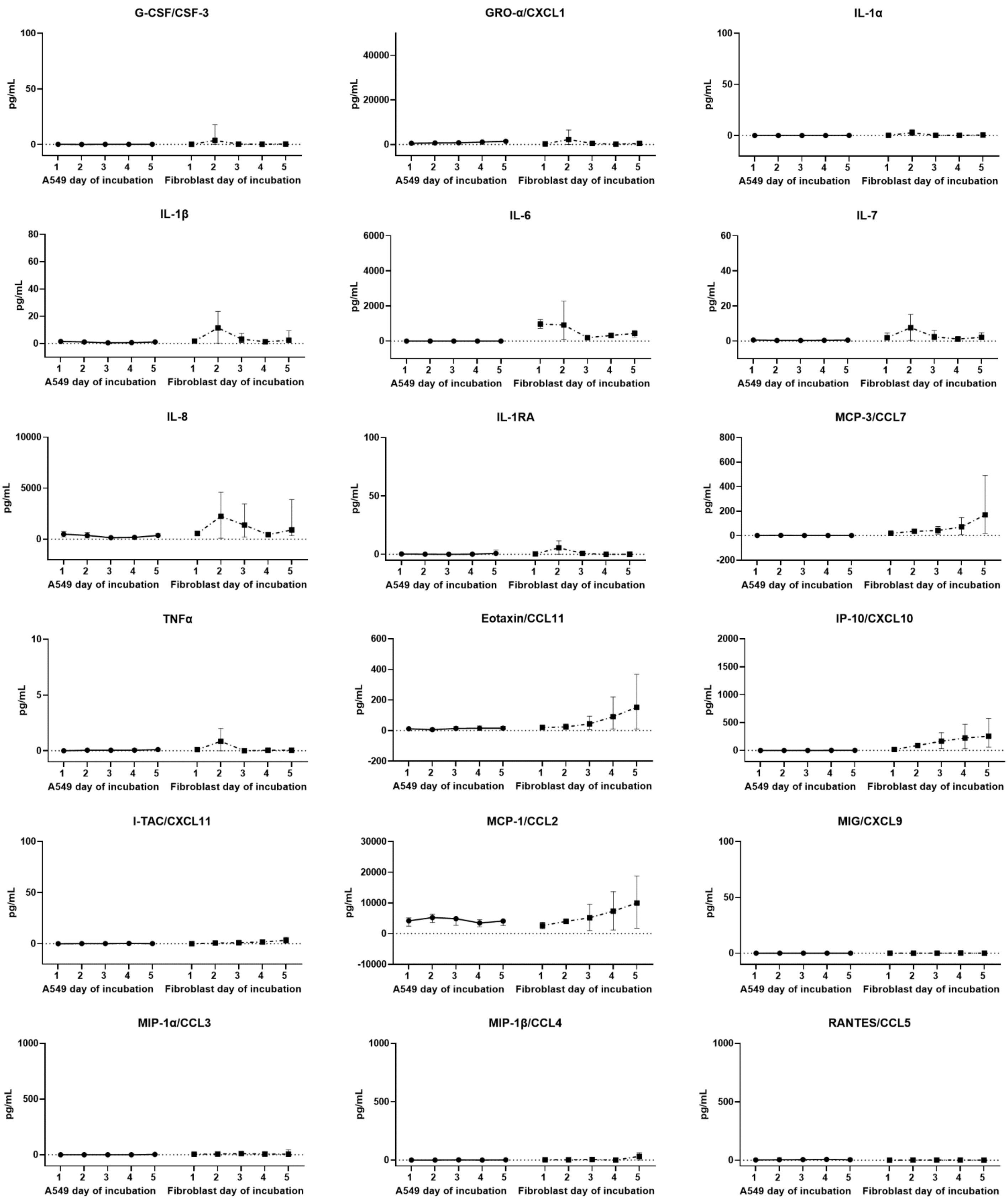

Supplementary Figure 2. Cytokine and chemokine concentrations measured in cell culture supernatants from A549s or fibroblasts every 24 hours after mock infection. Values are represented as median concentration (pg/mL) and interquartile range of concentrations in mock-infected cultures at each time point. The culture medium was almost completely replaced on each day of the assay, such that data represent cytokine secretion per day as opposed to cumulative secretion. Assays were performed in duplicate in 2 independent experiments.

Supplementary Figure 3

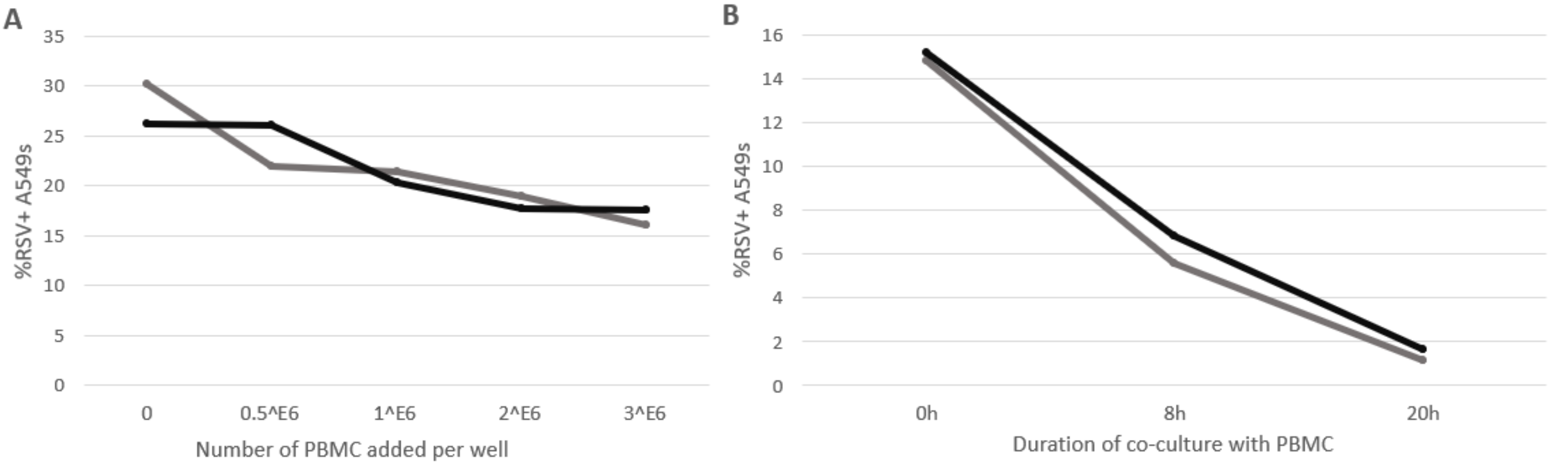

Supplementary Figure 3. Addition of PBMCs from healthy adult donors to RSV-infected respiratory epithelium attenuates the RSV infection. A) A549 cells were grown in 24 well plates at 100,000 cells/well and infected with RSV at MOI 0.6. Varying numbers of PBMC were added to the culture and incubated for 20 hours, then cells were collected and proportion of RSV-infected A549s were measured using flow cytometry. B) A549 cells were grown in 24 well plates at 100,000 cells/well and infected with RSV At MOI 0.3. PBMC (3^E6) were added to the culture and incubated for either 8 or 20 hours, then cells were collected and proportion of RSV-infected A549s were measured using flow cytometry. Assays were performed in duplicate in 1 experiment.

Supplementary Figure 4.

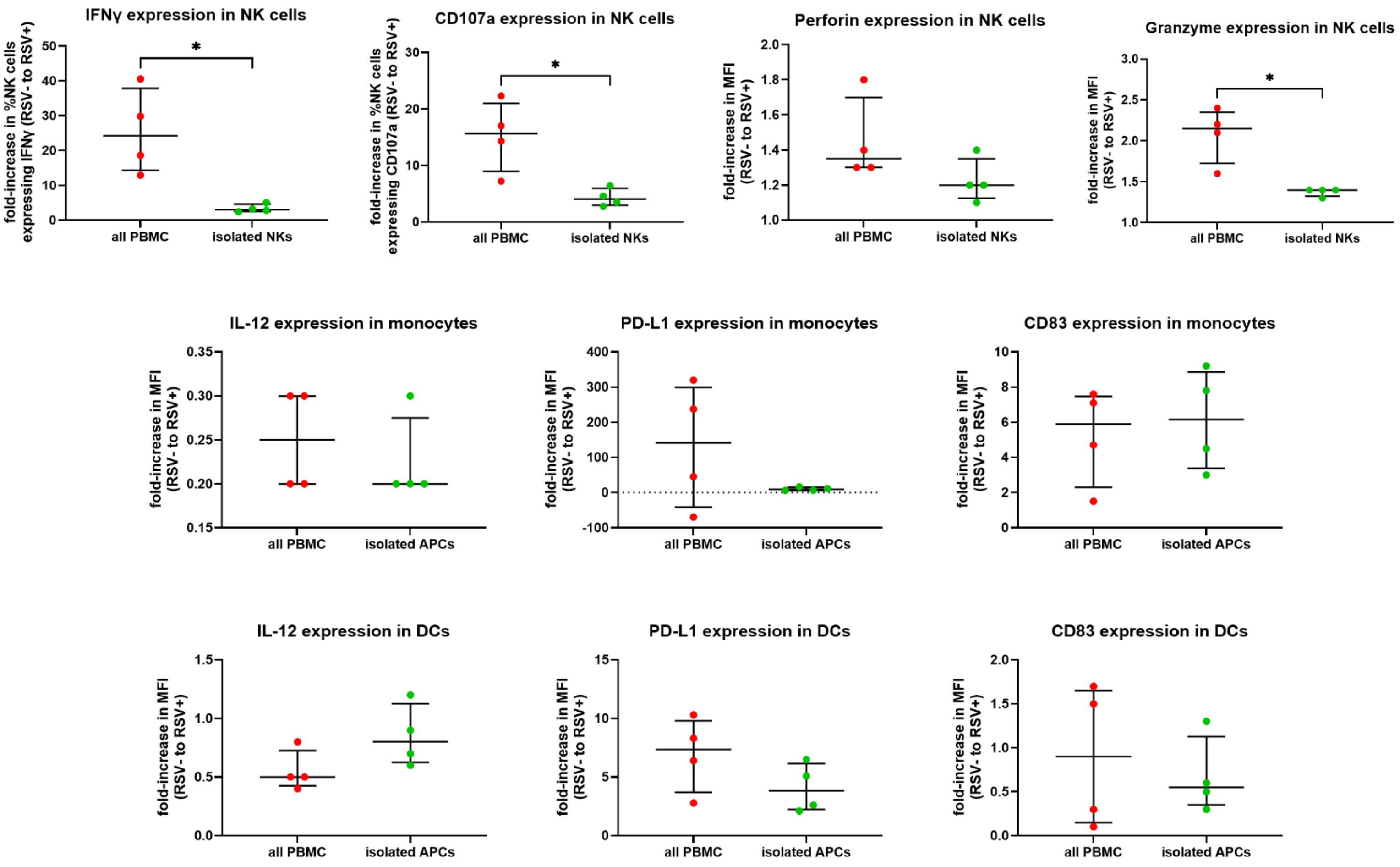

Supplementary Figure 4. Fold-change in expression of markers of activation, maturation, and degranulation among healthy adult innate immune cells after exposure to RSV-infected vs. mock-infected respiratory epithelial cells. Healthy adult PBMCs were enriched for either natural killer cells or antigen-presenting cells using magnetic bead-based negative selection isolation kits. The responses of these cell types were compared when they were added to a co-culture with RSV-infected respiratory epithelial cells in isolation versus with other PBMCs from the same donor. N=4 donors were included in each experiment. Significant differences are indicated by asterisks, where * represents p=0.01-0.05.

Supplementary Figure 5.

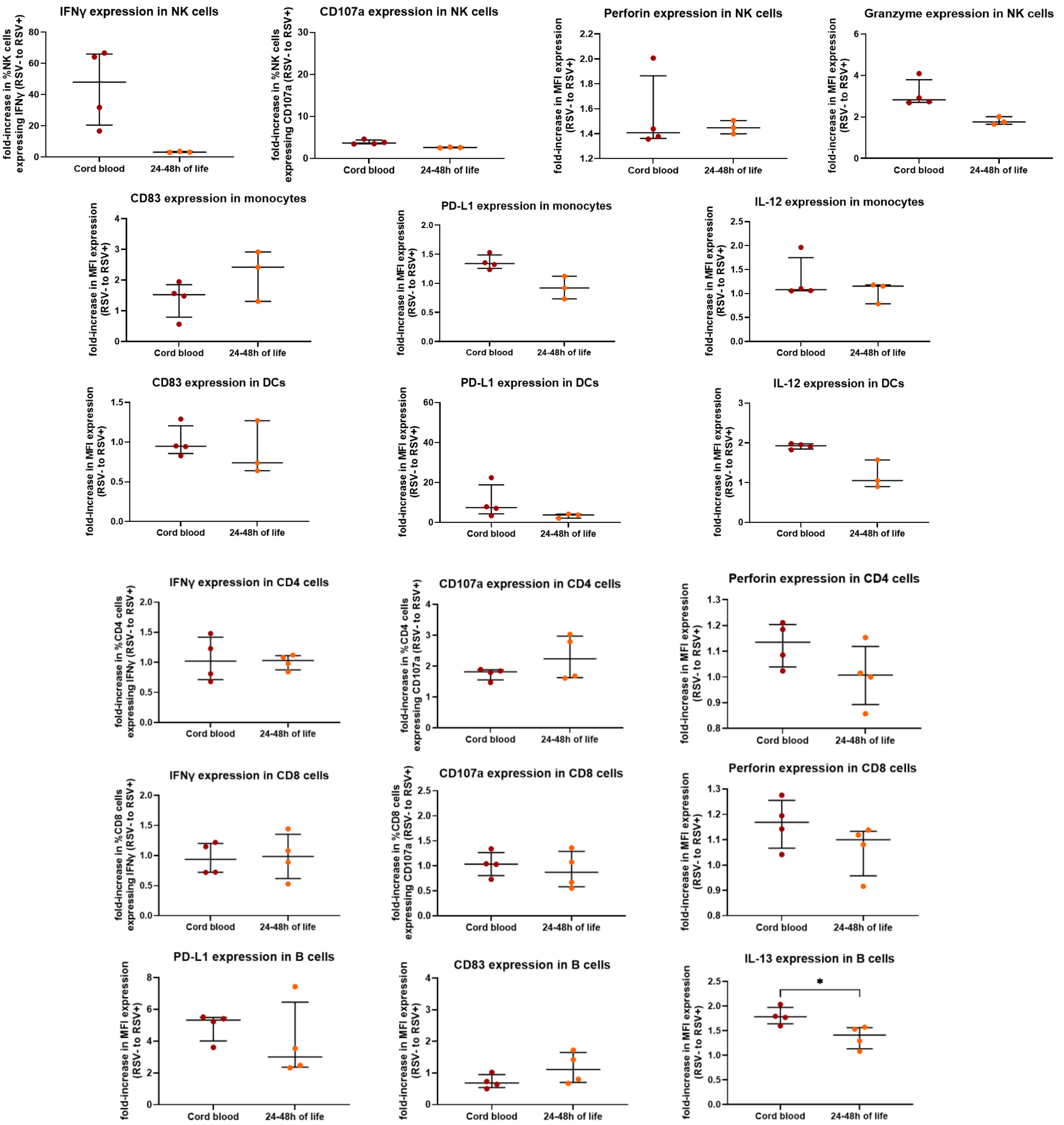

Supplementary Figure 5. Fold-change in expression of markers of activation, maturation, and degranulation among cord and peripheral blood mononuclear cells from infants 24-48 hours old after exposure to RSV-infected vs. mock-infected respiratory epithelial cells. N=4 participants were included at each age; all samples were run in the same experiment. Significant differences are indicated by asterisks, where * represents p=0.01-0.05.

Supplementary Table I. Demographic characteristics of infant and child blood donors

Infant/Child Group	Total N	N(%) used for innate panel	N(%) used for adaptive panel	N(%) female	Age at blood draw (median, range)	Race/Ethnicity (%)
Birth (cord blood)	4	4 (100%)	4 (100%)	3 (75%)	0 day (0-0 days)	Black Non-Hispanic 75%<br>White Non-Hispanic 25%
24-48h of life	7	3 (43%)	4 (57%)	4 (57%)	1 day (1 day-1 day)	Black Non-Hispanic 14%<br>Hispanic 71%<br>White Non-Hispanic 14%
12-18mo RSV seronegative	4	4 (100%)	4 (100%)	1 (25%)	15 months (14-18 months)	Asian 25%<br>Hispanic 75%
12-18mo RSV seropositive	4	4 (100%)	4 (100%)	2 (50%)	16 months (15-17 months)	Black Non-Hispanic 50%<br>Hispanic 50%
